# Effect of Electroacupuncture at Wushu Acupoints of the Cardiopulmonary Meridian on the Autophagy in Rats with Acute Myocardial Ischemia

**DOI:** 10.1155/2022/2114517

**Published:** 2022-03-21

**Authors:** Chao Zhu, Shengbing Wu, Xin Wu, Meiqi Zhou, Kun Wang, Shuai Cui, Jie Zhou

**Affiliations:** ^1^Graduate School, Anhui University of Chinese Medicine, Hefei, Anhui, China; ^2^Key Laboratory of Xin'an Medicine, Ministry of Education, Anhui University of Chinese Medicine, Hefei, Anhui, China; ^3^Bozhou Institute of Chinese Medicine, Anhui Academy of Traditional Chinese Medicine, Bozhou, Anhui, China; ^4^Institute of Acupuncture and Meridian, Anhui University of Chinese Medicine, Hefei, Anhui, China; ^5^Zhejiang Chinese Medical University, Hangzhou, Zhejiang, China

## Abstract

Wushu acupoints are the five acupoints distributed below the human elbow and knee joint. They are all located on the same meridian and divided into five categories: Jing, Ying, Shu, Jing, and He. It has been shown that electroacupuncture (EA) at Shenmen point of heart meridian can improve acute myocardial ischemia (AMI) early. However, it is still unclear if all the Wushu acupoints of the heart meridian can improve AMI. Hence, this study emphasizes Wushu acupoints of heart meridian, compares them with Wushu acupoints of lung meridian, and studies the therapeutic effect of EA at Wushu acupoints on AMI and its possible mechanism. It also discusses the specificity of the heart meridian to heart disease. The AMI model is established by ligation of the left anterior descending coronary artery. The detection methods like the physiological recorder, TTC staining, ELISA, and so forth were used to determine the ECG, myocardial infarct size, serum myocardial enzymes, and myocardial tissue-related protein expression in rats. The heart rate (HR) and ST segment along with creatine kinase (CK), creatine kinase isoenzymes (CK-MB), lactate dehydrogenase (LDH), and myocardial infarctions increased after the induction with AMI. Furthermore, the expressions of PINK1 and Parkin protein also showed an increase. However, EA at Wushu acupoints in the heart meridian can reverse the above changes, whereas EA at the lung meridian exhibits limited effect. It is depicted that the heart meridian has a relatively specific relationship with the heart in a diseased state.

## 1. Introduction

Acute myocardial ischemia (AMI) is a relatively common disease with severe myocardial damage. Changes in coronary flow cause AMI, resulting in an imbalance in oxygen demand between the blood and myocardium. Recently, it has become one of the key reasons for the increase of morbidity and mortality globally. Furthermore, the incidence rate of women is higher than that of men [1–3]. Studies at home and abroad have verified that acupuncture has a good therapeutic effect on myocardial ischemia [4, 5]. Acupuncture at Neiguan (PC 6), Shenmen (HT 7), and Lieque (LU 7) played a part in regulating the core mass, neurotransmitters, autonomic nervous activity, cardiac bioactive substances, ECG, and myocardial infarction size of AMI patients [6–11]. Additionally, in previous animal experiments, relevant researchers compared a single acupoint in the heart meridian with a single acupoint in the lung meridian to examine the difference in effects on AMI [12].

Wushu acupoints are acupoints present in the meridians and collaterals of the body and used in traditional Chinese medicine. They are distributed below the elbow and knee joint and are divided into five categories: Jing, Ying, Shu, Jing, and He. The Wushu acupoints of the heart meridian include the following: Jing-Shaochong, Ying-Shaofu, Shu-Shenmen, Jing-Lingdao, and He-Shaohai. At the same time, the Wushu acupoints of the lung meridian are as follows: Jing-Shaoshang, Ying-Yuji, Shu-Taiyuan, Jing-Jingqu, and He-Chize. The clinical application of Wushu acupoints is widespread, but each has its own emphasis. At present, there are no reports available on animal research of Wushu acupoints. Therefore, in this experiment, Wushu acupoints were used as the treatment point. A comparison between heart meridian and lung meridian was used to observe the effects of acupuncture at both heart and lung acupoints on the heart rate (HR), myocardial infarct size, the expression of myocardial enzymes, and autophagy proteins in AMI. Finally, we also discussed the specificity of the heart meridian to the heart.

**Figure 1:**
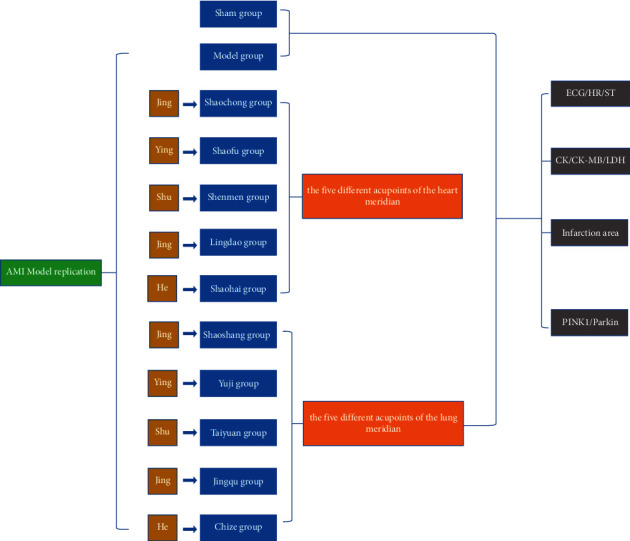
The flow diagram of the experiment.

The experimental process and group details are shown in Figure 1.

## 2. Materials and Methods

### 2.1. Animals and Groups

108 specific-pathogen-free Sprague Dawley (SD) rats (190–230 g) were provided by Pizhou Dongfang Aquaculture Co., Ltd. [animal license number SCXK (SU) 2017–0003]. A total of 120 SD male rats were prepared in advance. During the experiment, 12 rats died due to improper operation, excessive anesthesia, and euthanasia. Hence, 108 SD rats were involved in the experiment. SD rats were placed in the animal feeding room of Anhui University of Chinese Medicine for one week in a natural light environment and were fed food and water daily. The temperature in the cage was set at 19–21 degrees Celsius, and the relative humidity was maintained at 40–60%. To effectively carry out this experiment, we included nine rats in the sham group. The rest were included into the model group according to a random number table.

Further, the rats were divided into five acupoint groups in heart meridian (Jing-Shaochong, Ying-Shaofu, Shu-Shenmen, Jing-Lingdao, and He-Shaohai groups) and five acupoint groups in lung meridian (Jing-Shaoshang, Ying-Yuji, Shu-Taiyuan, Jing-Jingqu, and He-Chize group). A total of nine rats were included in each group. The treatment of animals during the experiment strictly abides by the ethical principle of welfare of experimental animals in the Research and Experiment Center of the Anhui University of Chinese Medicine.

### 2.2. Main Reagents and Instruments

The following reagents were used in this study: enzyme-linked immunoassay kit for creatine kinase (CK), creatine kinase isoenzyme (CK-MB), and lactate dehydrogenase (LDH) (Shanghai Jianglai Biotechnology Co., Ltd.); 2,3,5-triphenyl tetrazolium chloride (TTC) staining solution (Meilun Biotechnology Co., Ltd.); BCA protein quantitation kit (Thermo Fisher Scientific); PINK1, Parkin antibody (Abcam); *β*-actin (Beijing Zhongshan Jinqiao Company Biotechnology Co., Ltd.); goat anti-rabbit HRP-labeled secondary antibody (ZSGB-BIO); luminescent liquid (MILLIPORE); and PBS buffer (Shanghai Kearton Biotechnology Co., Ltd.).

The following instruments were used in this study: PowerLab 16 physiological recorder (AD Instruments, Australia); R500 universal small animal anesthesia machine (Shenzhen Ruiwode Life Technology Co., Ltd.); microplate reader (Shenzhen Redu Life Science Co., Ltd.); high-speed desktop refrigerated centrifuge (Anhui Jiawen Instruments Co., Ltd.); Hwato brand acupuncture needle (Beijing Luoya Shanchuan Medical Instrument Co., Ltd.); electronic acupuncture therapeutic apparatus (Suzhou Medical Appliance Factory Co., Ltd.); 37-degree incubators; electrophoresis instrument (BIO-RAD Co., Ltd.); and electrokinetic instrument (Dalian Jingmai Technology Co., Ltd.).

### 2.3. Animal Model

**Figure 2:**
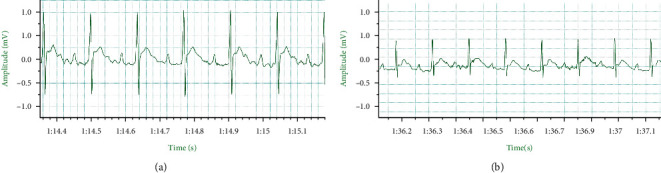
ECG before and after modeling in rats. (a) The ECG before modeling in rats. (b) The ECG after modeling in rats.

The SD rats were placed in a general anesthesia induction box for small animals to be induced with isoflurane at 5% concentration and then fixed on the rat board in the supine position. The anesthesia state was maintained using isoflurane at 2% to 3%. The skin was prepared at the heart of the chest for incision. Deep and shallow muscles of the chest were separated bluntly. Hemostatic forceps were used to open the 4th and 5th intercostal space and extrude the heart. The left anterior descending branch of the coronary artery was ligated using suture needle No.6–0 with line. The whitened myocardial tissue at the ligated part was visible to the naked eye, and then the heart was reset. Residual air trapped in the thoracic cavity was extruded. The thoracic cavity was sutured, and penicillin ointment was applied postoperatively to prevent infection. ECG was synchronously recorded, and the successful replication of the model was analyzed using ST-segment hunchback elevation in limb lead II and a high *T* wave. The threading was performed only at the sham group's left anterior descending coronary artery, and no ligation was executed. During the experiment, abnormal electrocardiograms and failure of modeling were excluded. ECG before and after modeling is revealed in Figure 2.

### 2.4. The Treatment

The sham and model groups were not treated with EA during the experiment. In the other treatment groups, the Wushu acupoints in the heart meridian and lung meridian were selected based on the acupoint positioning of rats in experimental Acupuncture Science. The disposable sterile acupuncture needles (specification parameter: Φ0.35 × 25 mm, Hwato brand) were inserted into the acupoints on the right side of the rat and subcutaneous part of the right hip. One end was connected to the positive electrode, whereas the other end was connected to the negative electrode. The current intensity and the current frequencies were 1 mV and 2 Hz/15 Hz, respectively. The treatment was conducted once a day, 30 min each time, for three successive days.

### 2.5. Method for Recording Rat Heart Rate and ST-Segment

The PowerLab 16 lead physiological recorder was used for analysis. The ECGs of rats before and after modeling were noted, and the failure in modeling was omitted. All groups were measured for 30 min again, after the final treatment in each treatment group. We recorded the limb II leads in all rats. The three-needle electrodes of the physiological recorder were divided into three parts and put into the subcutaneous layer of the right upper limb, left lower limb, and right lower limb in rats. The heart rate and ST-segment were recorded after the signal was stabilized.

### 2.6. Determination of Serum CK, CK-MB, and LDH

After the EA treatment, all rats were anesthetized with isoflurane, and 3 mL of blood was collected from the abdominal aorta into a 5 mL blood collection tube. The blood samples were centrifuged in a 4°C centrifuge at 3500 rpm for 15 min after standing for 1 h. The supernatant was collected and placed in a –80°C refrigerator for subsequent use; CK, CK-MB, and LDH contents were measured according to the manufacturer's instruction after the ELISA kits were balanced for 30 min at room temperature.

### 2.7. Measurement of Myocardial Infarction Area by TTC Staining

The myocardial tissue of each group was taken out after the collection of blood from the abdominal aorta. The blood on the tissue surface was washed with 4°C normal saline and stored in the –20°C refrigerator. The myocardial tissue was removed after 20 min and sliced with a blade, one at every 2 mm, and six slices were cut in total. The sections were immersed in TTC staining solution and incubated in the dark at 37°C for 30 min. Further, the sections were turned over once every 10 min to ensure that the myocardial tissues were fully in contact with the staining solution. A camera photographed the sections after incubation, and the infarction size was analyzed by ImageJ software.

### 2.8. Detection of the Expression Levels of Mitochondrial Autophagy Proteins PINK1 and Parkin in the Myocardial Tissue of Rats Using the Western Blotting Method

The myocardial tissues were weighed, cut into pieces, and added with the lysis solution in a proper proportion. They were then homogenized, lysed, and centrifuged at 12000 g for 15 min at 4°C. The supernatant was collected to quantify the protein, and a PAGE gel was used to separate the protein sample by electrophoresis at 120 V for 60 min. The protein was then transferred to the nitrocellulose (NC membrane) by a semidry transfer membrane at 25 V for 30 min. The antibody was diluted in PINK1 (1 : 1000) and Parkin (1 : 500) after blocking with 5% skimmed milk powder for 1 h at room temperature. After the antibody was added to the blocking solution and diluted to the required concentration, the same was incubated overnight with the membrane at 4°C. The TBST was performed three times, 5 min each time. The secondary antibody (1 : 10000) was added and incubated at 37°C for 1 h. The TBST was performed three times, 5 min each time, followed by ECL chemical coloration. The gray value of that band was analyzed using ImageJ software.

### 2.9. Statistical Analysis

All data are expressed as mean ± SD. SPSS 25.0 statistical software was used for analysis, and GraphPad Prism 8.0 was used for graph drawing. Using the Kruskal-Wallis univariate ANOVA test in nonparametric tests, comparisons between groups were conducted. *P* < 0.05 indicates that the difference was statistically significant.

## 3. Results

### 3.1. EA at Wushu Acupoints of Heart Meridian Inhibited the Level of HR and ST-Segment

Compared with the sham group, the HR and ST segments in the model group were significantly higher (*P* < 0.01). Compared with the model group, the HR and ST-segment decreased considerably in all Wushu acupoints in heart meridian groups (Shaochong, Shaofu, Shenmen, Lingdao, and Shaohai groups) (*P* < 0.01). Among the five lung meridian acupoints, only the Jingqu group showed a significant decrease in HR (*P* < 0.01) and ST-segment (*P* < 0.05), and the differences among the other groups were not statistically significant.

**Figure 3:**
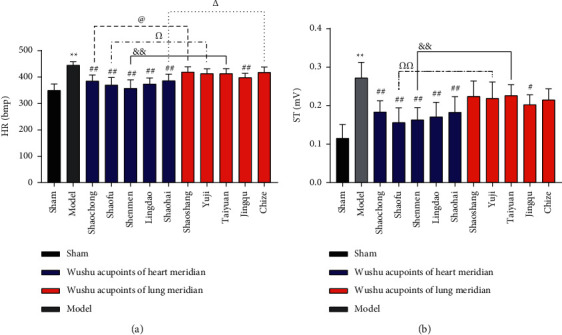
EA at Wushu acupoints of heart meridian inhibited the level of HR and ST-segment. The blue color in the histogram depicts the five different acupoints of the heart meridian. The red color in the histogram shows the five different acupoints of the lung meridian, such as (a) the HR histogram and (b) the ST histogram. Compared with the sham group, HR (*P* < 0.01) and ST (*P* < 0.01) were considerably increased in the model group. Compared with the model group, HR (*P* < 0.01) and ST (*P* < 0.01) decreased after EA at Shaochong, Shaofu, Shenmen, Lingdao, and Shaohai points of the heart meridian. Compared with the model group, HR (*P* < 0.01) and ST (*P* < 0.05) decreased after EA at the Jingqu points of the lung meridian. HR (*P* < 0.05) of Shaochong group was lower than that of Shaoshang group, HR (*P* < 0.05) and ST (*P* < 0.01) of Shaofu group were lower than those of Yuji group, HR (*P* < 0.01) and ST (*P* < 0.01) of Shenmen group were lower than those of Taiyuan group, and HR (*P* < 0.05) of Shaohai group was lower than that of Chize group. The number of rats in each group was nine,  ^*∗∗*^*P* < 0.01, ^#^*P* < 0.05, ##*P* < 0.01, ^@^*P* < 0.05, ^Ω^*P* < 0.05, ^ΩΩ^*P* < 0.01, ^&^*P* < 0.01, and ^△^*P* < 0.05.

Comparing the acupoints with the same cross section between heart meridian and lung meridian, the HR reducing effects of the Jing-acupoint Shaochong group and the He-acupoint Shaohai group in the heart meridian were better than those of the Jing-acupoint Shaoshang group and He-acupoint Chize group in the lung meridian (*P* < 0.05). The HR of AMI rats in the Shaofu group at Ying-acupoint in the heart meridian was lower than that in the Yuji group at Ying-acupoint in the lung meridian (*P* < 0.05). Furthermore, the ST-segment reduction was also better than that in the Yuji group (*P* < 0.01). The reducing degree of AMI HR and ST-segment in the Shenmen group of heart meridian was superior to that in the Taiyuan group of lung meridian (*P* < 0.01) (Figures 3(a) and 3(b)).

### 3.2. EA at Wushu Acupoints of Heart Meridian Reduced the Content of Serum CK, CK-MB, and LDH

**Figure 4:**
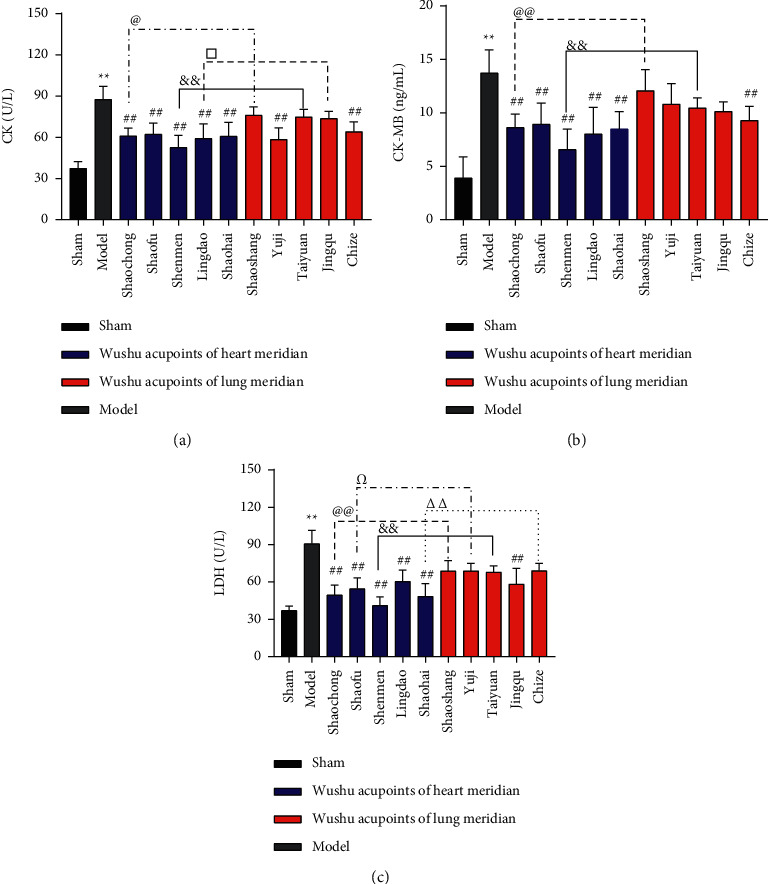
EA at Wushu acupoints of heart meridian decreased the content of serum CK, CK-MB, and LDH. The blue color in the histogram refers to the five different acupoints of the heart meridian. The red color in the histogram depicts the five different acupoints of the lung meridian. (a) The comparison of the serum CK levels in each group. (b) The comparison of the serum CK-MB levels in each group. (c) The comparison of the serum LDH levels in each group. Compared with the sham group, the levels of serum CK (*P* < 0.01), CK-MB (*P* < 0.01), and LDH (*P* < 0.01) increased considerably in the model group. Compared with the model group, the levels of serum CK (*P* < 0.01), CK-MB (*P* < 0.01), and LDH (*P* < 0.01) decreased after EA at the Shaochong, Shaofu, Shenmen, Lingdao, and Shaohai points of the heart meridian. In comparison with the model group, the levels of serum CK (*P* < 0.01) decreased after EA at the Yuji points and Chize points of the lung meridian, while the levels of serum CK-MB (*P* < 0.01) decreased after EA at Chize points of the lung meridian. The levels of serum LDH (*P* < 0.01) decreased after EA at Jingqu points of the lung meridian. The contents of serum CK (*P* < 0.05), CK-MB (*P* < 0.01), and LDH (*P* < 0.01) in the Shaochong group were lower than those in the Shaoshang group, and the contents of serum CK (*P* < 0.01), CK-MB (*P* < 0.01), and LDH (*P* < 0.01) in Shenmen group were lower than those in Taiyuan group, whereas the content of serum CK in Lingdao group (*P* < 0.05) was lower than that in Jingqu group. Finally, the content of serum LDH in the Shaofu group (*P* < 0.05) was lower than that in the Yuji group, and the content of serum LDH in the Shaohai group (*P* < 0.01) was lower than that in the Chize group. The number of rats in each group was nine,  ^*∗∗*^*P* < 0.01, ^##^*P* < 0.01, ^@^*P* < 0.05, ^@@^*P* < 0.01, ^Ω^P<0.05, ^&^*P* < 0.01, ^△△^*P* < 0.01, and ^□^*P* < 0.05.

Compared with the sham group, the model group's serum CK, CK-MB, and LDH contents increased significantly (*P* < 0.01). Compared with the model group, the contents of serum CK, CK-MB, and LDH in all five acupoints in heart meridian groups (Shaochong, Shaofu, Shenmen, Lingdao, and Shaohai) reduced considerably (*P* < 0.01). Among the five acupoints in lung meridian, only the serum CK content in the Yuji group was greatly reduced (*P* < 0.01), while the serum CK and CK-MB contents in the Chize group were reduced considerably (*P* < 0.01), and the serum LDH content in the Jingqu group was also significantly reduced (*P* < 0.01). The differences among the other groups were not statistically significant (Figures 4(a)–4(c)).

On comparing the Wushu acupoints with the same cross section of heart meridian and lung meridian, the reductions in the serum CK, CK-MB, and LDH contents in the heart meridian of the Jing-acupoint Shaochong group and the Shu-acupoint Shenmen group were superior to those in the lung meridian of Jing-acupoint Shaoshang group and the Shu-acupoint Taiyuan group (*P* < 0.05/0.01). The LDH content decreased greatly in that heart meridian. Ying-acupoint Shaofu group was superior to the lung meridian of Ying-acupoint Yuji group (*P* < 0.05). The reduction of serum CK content in the heart meridian of Jing-acupoints Lingdao group was greater than that in the lung meridian of Jing-acupoints Jingqu group (*P* < 0.05), and the reduction of serum LDH content in the heart meridian of He-acupoint Shaohai group was significantly superior to that in the lung meridian of He-acupoint Chize group (*P* < 0.01) (Figures 4(a)–4(c)).

### 3.3. EA at Wushu Acupoints of the Heart Meridian Reduced the Myocardial Infarction Size

**Figure 5:**
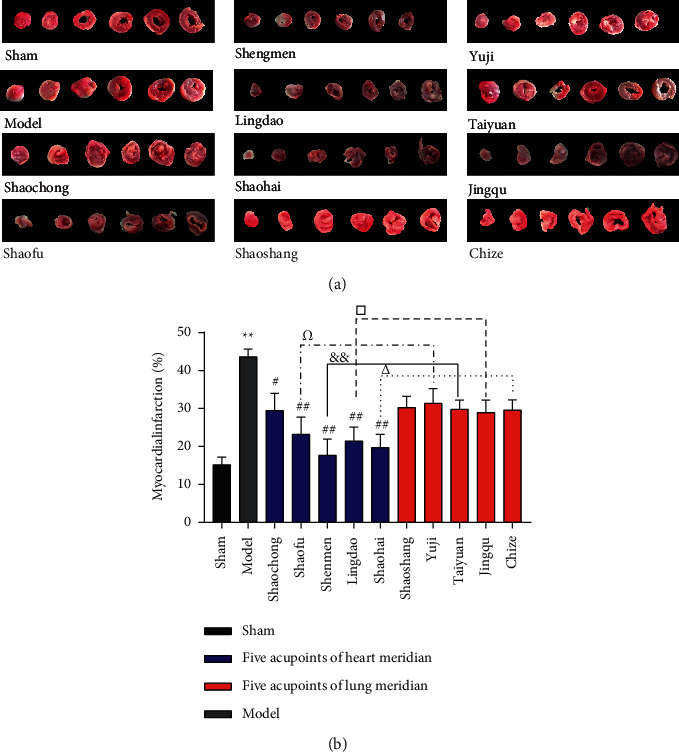
EA at the Wushu acupoints of the heart meridian reduced the myocardial infarction size. The blue color in the histogram reveals the five different acupoints of the heart meridian. The red color in the histogram shows the five different acupoints of the lung meridian. (a) TTC staining picture of myocardial tissue in each group. (b) The infarction size of the heart of AMI rats. The myocardial infarction size in the model group increased significantly (*P* < 0.01) when compared with the size in the same group. EA at Shaochong (*P* < 0.05), Shaofu (*P* < 0.01), Shenmen (*P* < 0.01), Lingdao (*P* < 0.01), and Shaohai (*P* < 0.01) of the heart meridian decreased the size of the myocardial infarction when compared with the model group. The ischemic area in Shaofu group (*P* < 0.05) was smaller than that in the Yuji group, while the ischemic area in Shenmen group (*P* < 0.01) was smaller than that in the Taiyuan group. Finally, the ischemic area in Lingdao group (*P* < 0.05) was smaller than that in the Jingqu group, and the ischemic area in Shaohai group (*P* < 0.05) was smaller than that in the Chize group. *n* = 6;  ^*∗∗*^*P* < 0.01, ^#^*P* < 0.05, ^##^*P* < 0.01, ^Ω^*P* < 0.05, ^&^*P* < 0.01, ^△^*P* < 0.05, and ^□^*P* < 0.05.

The ischemic area of myocardial tissue was grayish-white, and the nonischemic area was red or purplish-red, as shown in Figure 5(a).

Compared with the sham group, the myocardial infarction area in the model group was amplified significantly (*P* < 0.01). In comparison with the model group, the myocardial infarction area of rats in the Shaochong group reduced (*P* < 0.05), while those in Shaofu, Shenmen, Lingdao, and Shaohai groups decreased significantly (*P* < 0.01). There was no major difference in the myocardial infarction among the acupoint groups of the lung meridian. Comparing the acupoints with the same cross section of heart meridian and lung meridian, the Shenmen group via the heart meridian was superior to the Taiyuan group via the lung meridian (*P* < 0.01). The Shaofu, Lingdao, and Shaohai groups via the heart meridian were better than the Yuji, Jingqu, and Chize groups via the lung meridian (*P* < 0.05), as shown in Figure 5(b).

### 3.4. EA at Some Acupoints Decreased the Protein Expression of PINK1 and Parkin in Myocardial Tissue

**Figure 6:**
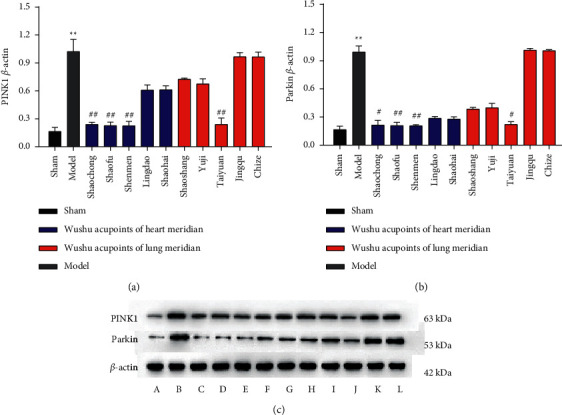
EA reduces the expression of PINK1 and Parkin protein in myocardial tissue of AMI rats. The blue color in the histogram indicates the five different acupoints of the heart meridian. The red color in the histogram depicts the five different acupoints of the lung meridian. (a) The PINK1 protein level. (b) The Parkin protein level. The levels of PINK1 and Parkin protein were considerably greater in the model group (*P* < 0.01) compared to the sham group. EA at Shaochong (*PP* < 0.01), Shaofu (*P* < 0.01), and Shenmen (*P* < 0.01) of heart meridian and Taiyuan (*P* < 0.01) of lung meridian showed reduced expression of PINK1 protein; EA at Shaochong (*P* < 0.05), Shaofu (*P* < 0.01), and Shenmen (*P* < 0.01) of heart meridian and Taiyuan (*P* < 0.01) of lung meridian also reduced the expression of Parkin protein. (c) The protein expression band diagram. *A* = sham group, *B* = model group, *C* = Shaochong group, *D* = Shaofu group, *E* = Shenmen group, *F* =Lingdao group, *G* = Shaohai group, *H* = Shaoshang group, *I* = Yuji group, *J* = Taiyuan group, *K* = Jingqu group, *L* = Chize group. *n* = 3;  ^*∗∗*^*P* < 0.01, ^#^*P* < 0.05, and ^##^*P* < 0.01.

Compared with the sham group, the protein expression levels of PINK1 and Parkin in the myocardial tissue of the model group increased (*P* < 0.01). Compared with the model group, the protein expression levels of PINK1 and Parkin in the Shaofu group and Shenmen group of the Wushu acupoints in the heart meridian decreased (*P* < 0.01). Further, the expression level of Parkin in the Shaochong group decreased significantly (*P* < 0.05), and the expression level of PINK1 also showed a reduction (*P* < 0.01). The Parkin level was reduced in the Taiyuan group (*P* < 0.05), and the PINK1 level decreased significantly (*P* < 0.01) among the Wushu acupoints in the lung meridian. Still, no major differences among the other groups were observed (Figures 6(a)–6(c)).

Moreover, there was no statistical significance in the difference between the heart meridian and lung meridian at the same cross-sectional acupoints.

## 4. Discussion

Wushu acupoints are the points and treatment points of human diseases. At present, there are few experimental studies on Wushu acupoints at home and abroad. Studies on meridians or acupoints mostly focus on the comparison of a single meridian or single acupoint with nonmeridian nonacupoints, because parameters such as meridian (or the size and depth of meridian acupoints) are not described in detail, and it is difficult to define the differences between meridians and nonmeridians. This study surpassed this point by comparing the heart meridian of *Shaoyin* in hand with the lung meridian of *Taiyin* in hand, adopting the comparison of “meridians–meridians,” taking the Wushu acupoints of the two meridians as the focus, and selecting the acupoints with the same cross section to observe the differences in intervention effects, thus avoiding the problem of meridian-nonmeridian definition. It was possible to more closely study the intervention effects of meridians rather than acupoints in heart meridian on such diseases as myocardial ischemia and prove the specificity of meridian therapy for diseases. In addition, the heart meridian and lung meridian of human body are short in route and single in function, which can reduce the experimental errors caused by various factors to a certain extent.

Our earlier studies displayed that EA at Shenmen can reduce AMI rats' heart rate, ST-segment, and myocardial infarction area and relieve myocardial injury. In this study, the HR, ST-segment, and myocardial infarction area were amplified after myocardial injury in rats, which showed that the heart function was damaged. After EA at Shenmen, all the above indexes decreased, proving that myocardial injury increased following the results of previous studies. Additionally, this study revealed that the acupoints of Jing, Ying, Shu, Jing, and He in the heart meridian exhibited regulatory effects on HR, ST-segment, serum CK, CK-MB, LDH content, and myocardial infarct size of AMI rats. There were individual acupoints in Wushu acupoints of lung meridian that could exert the effects, but the number of effective acupoints was smaller than that in the heart meridian. As per the results of this study, the impact of acupoints in the heart meridian on the enhancement of different indicators in rats with AMI was better than that in the lung meridian. This can provide some evidence for the specificity of heart meridian acupoints in treating heart diseases to some extent.

Serum CK, CK-MB, and LDH are the sensitive indicators for early diagnosis of AMI and are the representative serum biomarkers of myocardial ischemia and necrosis [13–15]. CK is widely present in the cytoplasm of the myocardial cells. Once the myocardial injury occurs, CK is released into the bloodstream, which sharply increases the content of CK in the blood [16], revealing high specificity. CK-MB, one of CK, also exists in the myocardium, and an increase in its content reflects the scope of ischemic necrosis [17, 18]. LDH is a key myocardial functional enzyme [19]. After myocardial damage, LDH will leak from the inside to the outside of the cells, and its quantity and quality will directly affect the body's energy metabolism. The finding of the LDH content has an important reference value for the clinical diagnosis of myocardial damage [20]. In this study, after the AMI rat model was established, the CK, CK-MB, and LDH contents increased in the model group, showing that myocardial ischemia and necrosis had occurred. The above indicators were reduced to different degrees after EA at the Wushu acupoints of the heart meridian, signifying that EA at the Wushu acupoints of the heart meridian could relieve the AMI state, regulate body energy metabolism, and improve the damaged myocardium. Moreover, only limited Wushu acupoints in the lung meridian affected the serum index, and the result was worse than that of the acupoints in the heart meridian. Besides, all the Wushu acupoints in the heart meridian could improve the serum CK, CK-MB, and LDH contents, so it could be deduced that the acupoints in the heart meridian exhibited specificity for the treatment of myocardial ischemia.

Autophagy is an extremely conservative lysosomal degradation pathway of the eukaryotic cells, which can degrade damaged organelles, intracellular invasive microorganisms, long-lived proteins, and so forth [21]. It is a self-protecting behavior during the process of growth and development of the body. Reports have shown that autophagy is closely related to cardiovascular disease [22]. Mitochondrial autophagy is a targeted phenomenon that can specifically recognize and degrade damaged mitochondria. It plays a key role in maintaining the stability of the intracellular environment and its functional state [23, 24]. Activating the mitochondria appropriately during AMI can protect cardiomyocytes and maintain mitochondrial homeostasis. Still, excessive and long-term upregulation of autophagy will disorganize the mitochondria, induce cardiomyocyte apoptosis, and aggravate the myocardial injury [25, 26]. Therefore, inhibition of autophagy may be a key point to improve myocardial injury.

The PINK1/Parkin pathway is one of the main mitochondrial-mediated autophagy pathways, in which PINK1 is an extremely conservative serine/threonine-protein kinase that is present abundantly in the myocardial tissue. Under physiological conditions, PINK1 will be transported from the mitochondrial outer membrane to the mitochondrial inner membrane and then cleaved and decomposed by presenilins-associated rhomboid-like (PARL) protein to maintain a low level [27]. However, mitochondria are damaged after injury to the body, and PINK1 cannot be transferred smoothly, cleaved, and degraded. Thus, they gather in a large amount in the mitochondrial outer membrane, leading to spatial structure changes and Parkin protein activation. The Parkin protein located in the cytoplasm is transferred to the mitochondria after activation, which in turn causes P62 and LC3 to bind to the mitochondrial matrix and induce mitochondrial autophagy [28–30]. In this study, the protein levels of PINK1 and Parkin in AMI rats were significantly increased as compared with the sham group, indicating that PINK1 and Parkin were activated and autophagy was exerted, confirming that PINK1/Parkin pathway was involved in the pathophysiological process of AMI. After EA, the protein levels of PINK1 and Parkin in the Shaochong, Shaofu, and Shenmen groups of heart meridian and the Taiyuan group of lung meridian were significantly decreased, indicating that autophagy was inhibited and myocardial cell necrosis was reduced.

In conclusion, according to the number of effective acupoints used in the treatment of myocardial ischemia at the Wushu acupoints of the heart meridian and the superior effect exerted by it when compared with the same cross section of the lung meridian, it can be inferred that the treatment of myocardial ischemia at the Wushu acupoints in the heart meridian is much more specific than that in the lung meridian. The protective effect of EA at Wushu acupoints on AMI may be attained by inhibiting the PINK1/Parkin autophagy pathway.

## Data Availability

The analyzed data sets generated during the present study are available from the corresponding author upon reasonable request.

## Abbreviations

AMI: Acute myocardial ischemia
EA: Electroacupuncture
Wushu acupoints of heart meridian: Jing-Shaochong, Ying-Shaofu, Shu-Shenmen, Jing-Lingdao, and He-Shaohai
Wushu acupoints of lung meridian: Jing-Shaoshang, Ying-Yuji, Shu-Taiyuan, Jing-Jingqu, and He-Chize
HR: Heart rate
SD: Sprague-Dawley
CK: Creatine kinase
CK-MB: Creatine kinase isoenzymes
LDH: Lactate dehydrogenase
ECG: Electrocardiograph
TTC: Triphenyl tetrazolium chloride
PINK1: PTEN induced putative kinase protein 1
ST: ST-segment.
